# Roasted Tartary Buckwheat Bran as a Material for Producing Rutin-Rich Tea Beverages

**DOI:** 10.3390/plants10122662

**Published:** 2021-12-03

**Authors:** Takahiro Noda, Koji Ishiguro, Tatsuro Suzuki, Toshikazu Morishita

**Affiliations:** 1 Hokkaido Agricultural Research Center, NARO, Shinsei, Memuro, Kasai-gun, Hokkaido 082-0081, Japan; kuro@affrc.go.jp (K.I.); tosikazu@affrc.go.jp (T.M.); 2 Kyushu-Okinawa Agricultural Research Center, NARO, Suya, Koshi, Kumamoto 861-1192, Japan; tsuzu@affrc.go.jp; 3 Institute of Crop Science, NARO, Kamimurata, Hitachiomiya, Ibaraki 319-2293, Japan

**Keywords:** Tartary buckwheat, bran, grain, rutin, tea

## Abstract

Tartary buckwheat bran, a byproduct of buckwheat milling, is commonly treated as waste. The present study examined the rutin content during successive infusions of roasted Tartary buckwheat bran and grain to develop a functional Tartary buckwheat tea. Samples (6 g) of roasted Tartary buckwheat bran and grain were rinsed with 300 mL of hot water (>95 °C) for 0.5 min. For the first infusion test, the tea infusion sample of roasted Tartary buckwheat bran contained a distinctly higher amount of rutin (389 mg/L) than that of the roasted Tartary buckwheat grain (68 mg/L). Overall, rutin was more effectively extracted from roasted Tartary buckwheat bran, as compared to roasted Tartary buckwheat grain.

## 1. Introduction

Tartary buckwheat (*Fagopyrum tataricum* Gaertn.) is a significant pseudo-cereal consumed in many countries, including India, Nepal, China, and Japan. Tartary buckwheat grain is commonly processed into flour to manufacture food products such as noodles. Tartary buckwheat grain has a unique chemical profile with markedly high levels of bioactive components, especially rutin and quercetin-3-O-rutinoside. Rutin is well known to have antioxidative [1,2,3,4], anti-hypertensive [5], and α-glucosidase inhibitory activities [6]. The rutin content in Tartary buckwheat seeds is approximately 100-fold greater than that of common buckwheat seeds [2]. However, Tartary buckwheat seeds have extremely high rutinosidase activity, which hydrolyzes rutin to quercetin and rutinose within a few minutes after the addition of water to Tartary buckwheat flour [7,8]. To prevent rutin hydrolysis, we have developed a new Tartary buckwheat variety, ‘Manten-Kirari’, containing only trace amounts of rutinosidase [9,10]. The use of ‘Manten-Kirari’ enables us to facilitate the development of rutin-rich foods [11,12,13]. With conventional Tartary buckwheat variety ‘Hokkai T8’, with high rutinosidase activity, rutin was hydrolyzed almost completely in all foods tested [11,13]. Moreover, in a clinical study, the intake of rutin-rich noodles containing ‘Manten-Kirari’ reduced body weight, BMI (body mass index), and TBARS (2-thiobarbituric acid reactive substances) levels [14]. Tartary buckwheat bran, a byproduct of buckwheat milling, is usually discarded as waste, causing disposal issues. Buckwheat bran is well recognized for accumulating bioactive compounds, including rutin [15]. Our study has revealed that the rutin content of the ‘Manten-Kirari’ bran was five to ten times higher than that of flour [16]. Additionally, our more recent study has established a method to prepare roasted ‘Manten-Kirari’ bran with a high level of rutin, which is a promising source of rutin-rich raw materials for the making of health-beneficial foods [17]. Tartary buckwheat is partially marketed as grain for the manufacture of grain tea beverages due to its unique malty aroma [18]. The health benefits of Tartary buckwheat tea beverages may be associated with their rutin content [18]. The advantage of using roasted Tartary buckwheat bran for making tea is that roasted Tartary buckwheat bran has a higher level of rutin than Tartary buckwheat grain. The objective of our present research was to determine the rutin content in the aqueous extracts of roasted Tartary buckwheat bran and grain from a new variety, ‘Manten-Kirari’, during successive infusions.

## 2. Results and Discussion

**Figure 1:**
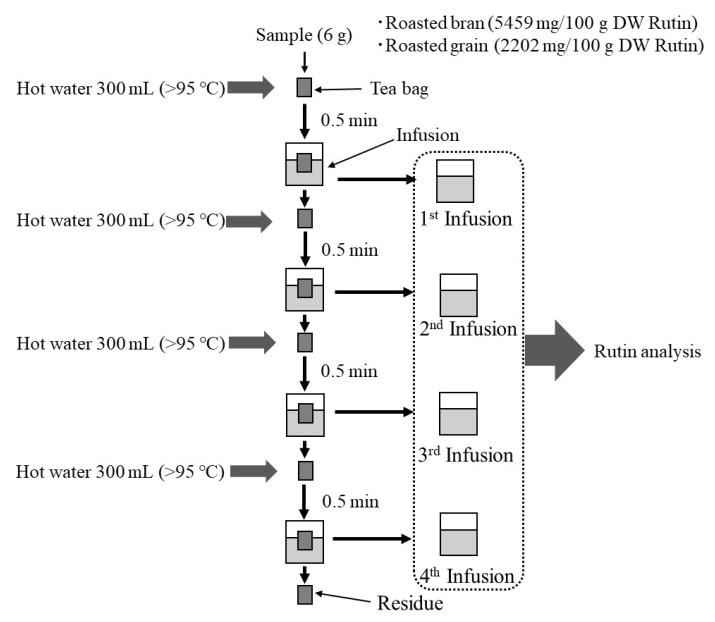
Flow diagram of rutin analysis of Tartary buckwheat bran and grain tea infusion samples.

In this study, roasted Tartary buckwheat bran and grain before infusion were first used to measure rutin content. The determined rutin content of roasted Tartary buckwheat bran and grain are presented in Figure 1. The rutin content of roasted Tartary buckwheat bran was as high as 5459 mg/100 g dry weight (DW). Our previous study reported that the rutin content of raw Tartary buckwheat bran varied from 3032 to 8649 mg/100 g DW [16]. Other studies also found similar values of rutin content in raw Tartary buckwheat bran [19,20,21]. Additionally, the optimum roasting condition caused very little change in the rutin content of Tartary buckwheat bran [17]. Namely, the rutin content of Tartary buckwheat bran before roasting was determined to be 5991 mg/100 g DW, while Tartary buckwheat bran after optimum roasting had a similar concentration of rutin (5459 mg/100 g DW). As compared to roasted Tartary buckwheat bran, roasted Tartary buckwheat grain had lower rutin content (2202 mg/100 g DW). We previously found that raw Tartary buckwheat grain had a higher rutin content (2825 to 2952 mg/100 g DW) [16]. This difference was presumably due to the cultivation conditions and/or the loss of rutin during roasting.

**Figure 2:**
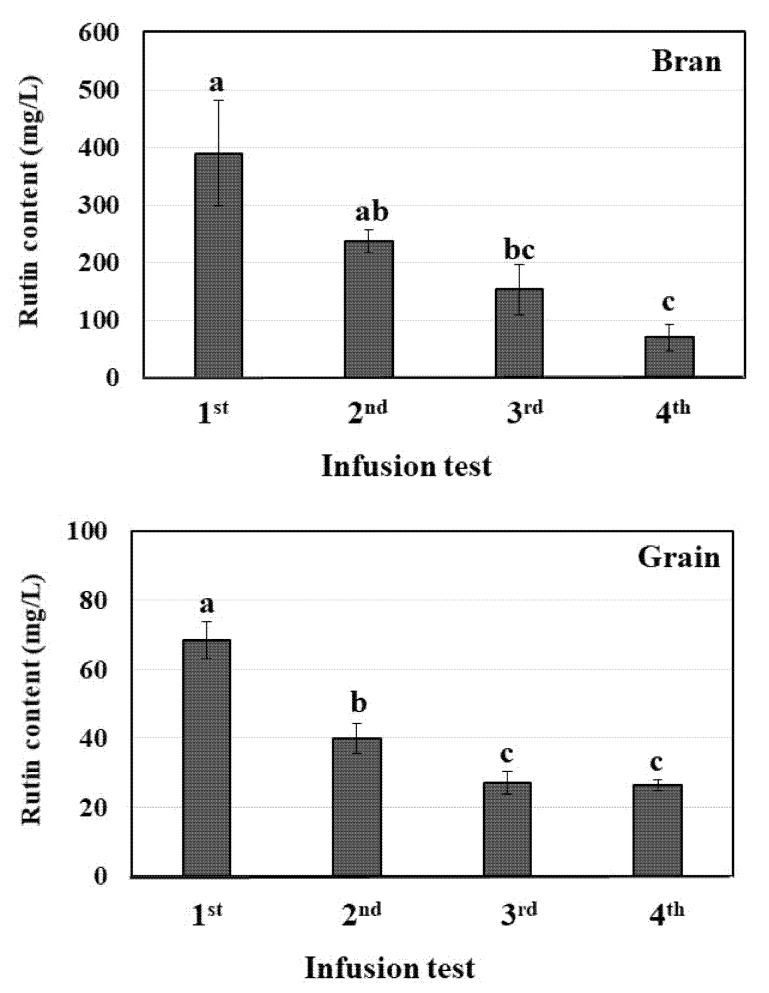
The rutin content of tea infusion samples of roasted Tartary buckwheat bran and grain in each infusion test. The data are averages ± the standard deviation of four determinations; the same letter within bran and grain indicates no significant difference at *p* < 0.05.

Although the solubility of rutin in cold water is unsatisfactory (0.13 g/L), its solubility in water increases sharply with temperature [22]. Thus, hot water treatment is a convenient, cheap, and environmentally friendly process for the extraction of rutin. Next, the first through fourth infusion tests of roasted Tartary buckwheat bran and grain were carried out using hot water (>95 °C). The rutin content of the tea infusion samples in each test was determined; the results are given in Figure 2.

**Figure 3:**
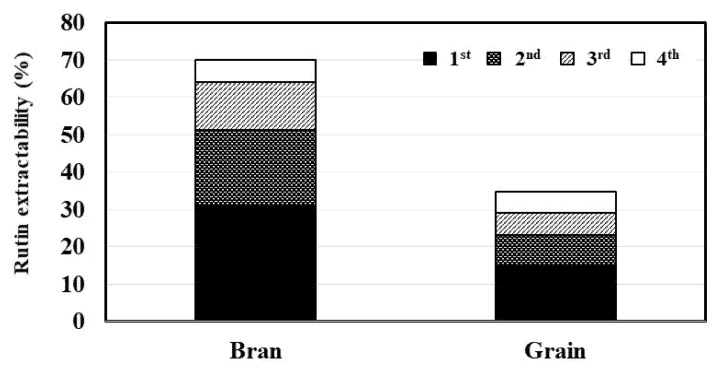
The rutin extractability of roasted Tartary buckwheat bran and grain in each infusion test. The data are averages of four determinations.

For the first test, the concentration of rutin (389 mg/L) in the tea infusion sample of roasted Tartary buckwheat bran was about six-fold higher than that of roasted Tartary buckwheat grain (68 mg/L). Repeated infusion clearly led to a reduction in the rutin content in tea infusion samples of roasted Tartary buckwheat bran. Consequently, for the fourth test, distinctly decreased rutin content was observed in the tea infusion samples of roasted Tartary buckwheat bran (70 mg/L). For roasted Tartary buckwheat grain, a manifest decline in the rutin content was found from the first to the third infusion test, whereas no significant difference in the rutin content (26.3 to 27.0 mg/L) was observed between the third and fourth infusion tests. Moreover, the rutin extractabilities of the tea infusion samples obtained from roasted Tartary buckwheat bran and grain were determined. For the second to fourth infusion tests, the sum of the rutin extractability of all tea infusion samples was calculated. As shown in Figure 3, the rutin extractability of roasted Tartary buckwheat bran for the first infusion test was twice as high (31.1%) as that of roasted Tartary buckwheat grain (14.7%). Improvements in the rutin extractability of roasted Tartary buckwheat bran and grain were found with increased infusion trials. The rutin extractability of roasted Tartary buckwheat bran for the fourth infusion test was distinctly higher (70.2%) than that of the roasted Tartary buckwheat grain (34.7%).

According to a recent review article of Suzuki et al. [23], the intake of Tartary buckwheat products with higher rutin content is beneficial for human health. For example, a double-blind clinical trial revealed that the two-week intake of rutin-fortified Tartary buckwheat cookies led to lower levels of total cholesterol and myeloperoxidase, an antioxidant marker, as compared to control cookies [24]. Another clinical trial showed that the intake of rutin-rich Tartary buckwheat noodles was effective for regulating body weight, presumably because of their antioxidant properties [14]. Therefore, consumers can possibly obtain rutin-derived health benefits from the intake of the Tartary buckwheat tea beverage described here.

Tartary buckwheat bran is an agricultural waste product of the buckwheat flour industry. Cho et al. [20] developed novel wheat-based products containing a rutin-enriched material prepared from Tartary buckwheat bran. Our results reveal that tea extract from roasted Tartary buckwheat bran has a clearly higher rutin content than that obtained from roasted Tartary buckwheat grain, which is commonly consumed in the form of tea beverages. Using Tartary buckwheat bran to make rutin-rich tea beverages will eliminate the disposal problem, while paving the way for a new functional food industry.

## 3. Materials and Methods

### 3.1. Materials

Tartary buckwheat bran from the variety ‘Manten-Kirari’ was purchased from Kobayashi Shokuhin Co., Ltd., Okoppe, Hokkaido, Japan. Roasted Tartary buckwheat bran was prepared as described previously [17]. One hundred grams of Tartary buckwheat bran was pretreated for 1 h at 105 °C, and was then treated for 10 min at 220 °C. Roasted Tartary buckwheat grain from the variety ‘Manten-Kirari’ was also purchased from Kobayashi Shokuhin Co., Ltd., Okoppe, Hokkaido, Japan; it is a commercial product used for tea beverages.

### 3.2. Infusion Test

Roasted Tartary buckwheat bran and grain tea samples were produced in infusion tests (first through fourth) as described in Figure 1. For the first infusion test, a 6 g sample of Tartary buckwheat bran or grain was inserted into a tea bag. Each tea bag was rinsed with 300 mL of hot water (>95 °C) for 0.5 min and then squeezed with tweezers. Repeated infusion tests were carried out to enhance the extractability of the rutin. For the second infusion test, the tea bag used for the first infusion was similarly rinsed with hot water and then squeezed. For the third and fourth infusion tests, the infusion process was repeated three and four times, respectively. Infusion tests were carried out four times. All tea infusion samples were collected and then stored at −20 °C until analysis.

### 3.3. Determination of Rutin Content

The tea infusion samples obtained above were analyzed for their rutin content. Additionally, roasted Tartary buckwheat bran and grain before infusion were also used for rutin analysis. The rutin content was measured by HPLC as described previously [8]. The total amount of rutin in roasted Tartary buckwheat bran and grain before infusion was regarded as the maximum extractible rutin and was defined as 100% rutin extractability.

### 3.4. Statistical Analysis

Averages of the rutin content of tea infusion samples were computed, and Tukey’s range tests were conducted to measure variations in rutin content among roasted Tartary buckwheat bran and grain tea samples of various infusions.

## 4. Conclusions

Tartary buckwheat bran is commonly regarded as waste in the buckwheat flour industry. Thus, the present study highlighted the development of roasted Tartary buckwheat bran tea using a new variety, ‘Manten-Kirari’, through successive infusions of roasted Tartary buckwheat bran for its effective utilization. Roasted Tartary buckwheat grain was also used as a control. As compared to those of roasted Tartary buckwheat grain, tea infusion samples of roasted Tartary buckwheat bran had distinctively higher concentrations of rutin. Consequently, roasted Tartary buckwheat bran can be preferably used in the production of rutin-rich tea beverages.

## Data Availability

Not applicable.
